# The Pathophysiological Association Between Obstructive Sleep Apnea and Glaucoma: A Current Update

**DOI:** 10.3390/jcm15135215

**Published:** 2026-07-03

**Authors:** Wojciech Mazurek, Łukasz Mazurek, Barbara Rękas-Mazurek, Marek Rękas

**Affiliations:** 1 Department of Ophthalmology, Military Institute of Medicine-National Research Institute, 04-141 Warsaw, Poland; 2 Department of Otorhinolaryngology, Faculty of Medicine and Dentistry, Medical University of Warsaw, 00-739 Warsaw, Poland; lukasz.mazurek1@wum.edu.pl

**Keywords:** glaucoma, normal-tension glaucoma, obstructive sleep apnea, chronic intermittent hypoxia, ocular perfusion pressure, lamina cribrosa, continuous positive airway pressure

## Abstract

Glaucoma is a chronic, progressive optic neuropathy and the second leading cause of irreversible blindness worldwide. Although elevated intraocular pressure (IOP) remains the principal modifiable risk factor, it is neither necessary nor sufficient for disease development. The literature indicates that systemic conditions such as obstructive sleep apnea (OSA) may contribute to its pathogenesis. The pathophysiology of glaucoma is supported by several theories, primarily the mechanical and vascular theories. This review describes the pathophysiological links between OSA and glaucoma considering current theories. The principal connecting mechanism appears to be chronic intermittent hypoxia and reduced ocular perfusion pressure, which trigger optic nerve head hypoxia, oxidative stress, and biomechanical remodeling of the lamina cribrosa. These processes interact within a vicious cycle that progressively compromises the metabolic support of optic nerve axons. The mechanisms described are particularly relevant to normal-tension glaucoma, which may be associated with OSA. Retinal nerve fiber layer thinning appears among the earliest markers of optic nerve vulnerability, whereas IOP and visual field changes are more variable. These observations underscore the clinical relevance of the OSA–glaucoma relationship and support a multidisciplinary approach incorporating routine ophthalmic screening for subclinical optic nerve damage.

## 1. Introduction

Glaucoma is a chronic and progressive optic neuropathy, which remains the second leading cause of irreversible blindness worldwide [1]. The prevalence among people after 40 ranges from 2.93% to 3.5% and increases with age, reaching 10% in people aged 90. It is estimated that by 2040, the global population of people with glaucoma is expected to increase to over 111 million [2,3,4]. This is clinically relevant because common chronic conditions, such as obstructive sleep apnea, diabetes mellitus, hypertension, hypothyroidism, or chronic obstructive pulmonary disease (COPD), are associated with an increased risk of developing glaucomatous optic neuropathy (GON) [5,6]. Early diagnosis and treatment of these conditions may have an influence on GON prevention.

Obstructive sleep apnea (OSA) is a sleep disorder defined by recurrent episodes of upper airway collapse during sleep, leading to intermittent hypoxia, hypercapnia, and sleep fragmentation. Despite its high prevalence, over 80% of cases remain undiagnosed [7,8]. In glaucoma, OSA may contribute to optic nerve damage through both indirect systemic effects and direct hypoxia-related mechanisms. Indirectly, OSA is a well-established risk factor for cardiovascular disease [9], which impairs ocular perfusion. Directly, recurrent episodes of intermittent hypoxia, oxidative stress, and increased sympathetic activity during sleep may raise vascular resistance, reduce ocular blood flow, and damage the optic nerve [10]. Importantly, the coexistence of these mechanisms in a frequently underdiagnosed condition may amplify their cumulative impact on optic nerve vulnerability.

Several studies have confirmed a significant association between OSA and glaucoma. However, the existing literature has largely focused on epidemiological data and structural ocular parameters. The pathophysiology of glaucoma itself remains incompletely understood, which makes characterizing its relationship with OSA particularly challenging. A deeper understanding of glaucoma pathophysiology is therefore essential to explain how OSA may contribute to optic nerve damage. To the best of our knowledge, no review has yet comprehensively examined this relationship in the context of current glaucoma pathophysiology concepts, several of which remain debated among ophthalmologists [5,11,12].

## 2. Pathophysiology of Glaucoma

The hallmark of glaucoma is the progressive degeneration of retinal ganglion cells (RGCs) and damage to the optic nerve head (ONH). This leads to axonal loss, optic disc cupping, and gradual visual field deterioration [1]. Although elevated intraocular pressure (IOP) (>21 mmHg) remains the most crucial modifiable risk factor, it is not required for disease development. Thus, normal-tension glaucoma (NTG) remains a subject of debate among ophthalmologists [13,14,15]. Population-based studies indicate that a significant proportion of primary open-angle glaucoma (POAG) occurs at normal IOP (NTG), ranging from 30.0% to 57.1% of POAG in white and African-descent populations and the majority of cases (up to ~92%) in East Asian populations [16].

The pathophysiology of glaucoma is not fully understood, and each type of glaucoma has a specific etiology. GON is primarily associated with increased intraocular pressure, which is regulated by aqueous humor (AH) dynamics. AH is produced by the ciliary processes of the ciliary body and is secreted into the posterior chamber at a rate of approximately 2–3 μL/min during the day, while its production decreases by about 50% at night. Aqueous humor flows from the posterior chamber of the eye through the pupil into the anterior chamber, where one of the main outflow pathways is located. Depending on age, aqueous humor drains from the eyeball largely through the trabecular meshwork outflow (conventional) or by the uveoscleral outflow (non-conventional) [17,18].

Disorders in the dynamics of aqueous humor affect IOP, which is significantly correlated with retinal ganglion cell degeneration [19]. On the other hand, increased IOP is not the single factor leading to glaucomatous neuropathy.

The literature identifies at least two primary pathophysiological theories, namely the mechanical and the vascular [20], both of which are essential to this paper. Furthermore, authors also mention the excitotoxicity theory, the neurotrophic factor deprivation theory, oxidative stress, and inflammation [5,21]. Although these concepts are often discussed separately, current evidence indicates substantial overlap between them in glaucomatous neurodegeneration.

The mechanical theory proposes that pressure on the optic nerve head inhibits axoplasmic flow in retinal ganglion cells, leading to apoptosis and necrosis. Moreover, increased IOP causes deformation of the lamina cribrosa (LC), which enhances the death of axons. In contrast, the vascular theory posits that hypoperfusion contributes to RGC apoptosis [22,23]. Notably, the direct factor in the vascular theory is not optic nerve hypoperfusion itself, but hypoxia.

Other theories include mitochondrial dysfunction, glutamate excitotoxicity, and neuroinflammatory processes. These mechanisms interact in a complex and synergistic manner, suggesting that glaucomatous neurodegeneration results from the convergence of multiple pathological pathways rather than a single dominant factor [24].

Notably, from the perspective of this paper, optic nerve hypoxia results from hypoxemia, insufficient blood supply, or the correlation between intraocular pressure and its impact on ocular perfusion pressure (OPP) [25,26,27].

### 2.1. Ocular Perfusion and Vascular Mechanisms in Glaucoma

Ocular perfusion pressure is a parameter that determines the ability of blood flow in the tissues of the eyeball. It is the difference between mean arterial pressure (MAP) and intraocular pressure (OPP = MAP − IOP) [27,28,29]. Many systemic disorders, such as hypertension, hypotension, diabetes, vascular disabilities, and OSA (importantly for the following article), lead to optic nerve hypoperfusion [30].

**Figure 1:**
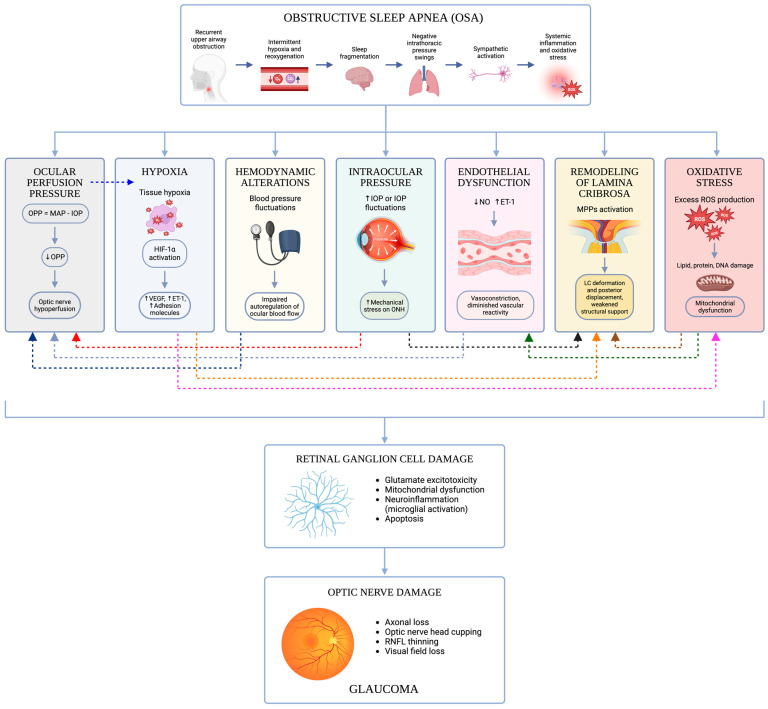
The vicious cycle of glaucoma pathogenesis in obstructive sleep apnea. Dashed lines indicate the relationships between factors. *RNFL-retinal nerve fiber layer; IOP-intraocular pressure; LC-lamina cribrosa; RGCs-retinal ganglion cells; OPP-ocular perfusion pressure; MAP-mean arterial pressure; MMPs-matrix metalloproteinases; ROS-reactive oxygen species* [27,33,34,35,36,37,38]. Created in BioRender. Mazurek, W. (2026) https://BioRender.com/mcqqfep (accessed on 30 May 2026).

As illustrated in Figure 1, the pathogenesis of glaucoma is determined by multiple interrelated factors. In the context of normal-tension glaucoma, the mechanical theory alone cannot explain the disorder [25,31,32].

Hypoperfusion decreases the MAP-to-IOP ratio, leading to hypoxia of the ONH and the degeneration of RGCs. Furthermore, the hypoxia induces oxidative stress, which promotes cell apoptosis. It was shown that an IOP above 40 mmHg or an OPP below 50 mmHg overwhelms the autoregulatory capacity of blood flow, leading to optic nerve ischemia [27,30,39].

Leske et al. [40] conducted a prospective cohort study as part of the Barbados Eye Studies (3222 participants without glaucoma at baseline). The study showed that 125 participants developed open-angle glaucoma (4.4%). The authors noticed an association with low IOP and glaucoma risk. Higher perfusion pressure was protective (RR, 0.66; 95% CI, 0.54–0.80 per 10 mmHg). Low perfusion pressure (<40 mmHg) more than doubled the risk of disease (RR 2.6; 95% CI: 1.4–4.6). These results show that vascular factors, especially reduced perfusion pressure, play a major role in the pathogenesis of glaucoma. This supports the vascular hypothesis as a complement to the classic IOP-dependent mechanism.

Wang et al. (2012) [41] performed a study using a glaucoma model in *Macaca mulatta*. The study included 9 subjects in whom experimental glaucoma (EG) was induced in one eye by causing laser damage to the trabecular meshwork, which led to an increase in IOP. The second eye of each subject served as a control, with no intervention performed. In the EG group, IOP was 30 ± 6 mm Hg, while in the control eyes it was 13 ± 2 mm Hg. Blood flow (BF) in the optic nerve head was assessed in both groups. In the glaucomatous eyes, a significant reduction in BF in the ONH of 22 ± 13% was observed (*p* = 0.003). A statistically significant thinning of the RNFL was also demonstrated compared with the control group, amounting to 42 ± 16% (*p* < 0.0001). This study demonstrated that elevated intraocular pressure reduces perfusion pressure and contributes to glaucomatous neuropathy.

Another study evaluating the association between OPP and glaucoma was the multicenter Egna-Neumarkt Study conducted by Bonomi et al. [42]. It included 4178 participants aged 40 or older. In individuals with low diastolic pressure (<50 mmHg), the risk was significantly increased (*p* < 0.01) compared with those with higher diastolic pressure. Furthermore, low OPP was associated with an increased incidence of glaucoma (OR 3.2; 95% CI: 1.4–7.1). Importantly, this association was independent of elevated IOP, suggesting that vascular factors may play a role independent of the pressure mechanism.

The Thessaloniki Eye Study performed by Topouzis et al. [43] included 232 subjects. The authors showed that decreased diastolic blood pressure (DBP) (<90 mmHg), particularly in patients receiving hypotensive therapy, was independently associated with a higher cup-to-disc ratio (CDR) (*p* = 0.02) and a smaller rim area (*p* = 0.01). However, no association was found for systolic blood pressure (SBP) (*p* > 0.05). Lower DBP remained an independent predictor of adverse structural changes in the ONH. Theoretically, this also suggests that a decrease in DBP may reduce perfusion to the optic nerve head, leading to structural changes. However, it is important that an increased CDR alone is not sufficient to diagnose glaucoma.

On the other hand, Quigley et al. [44] in the Proyecto VER population-based cross-sectional study (n = 4774) found no significant association with blood pressure. This shows that there are also some less common opposite results.

### 2.2. Biomechanics of the Optic Nerve Head

The optic nerve is divided into four segments: intraocular, intraorbital, intracanalicular, and intracranial. The intraocular segment is further subdivided into the (1) nerve fiber layer, (2) prelaminar, (3) laminar (lamina cribrosa), and (4) postlaminar portions [45,46,47].

The axons of RGCs in the lamina cribrosa (LC) are primarily metabolically dependent on diffusion from the LC blood vessels. Blood supply disturbances, such as reduced cerebral blood flow or hypoxia, cause remodeling of the LC and the peripapillary sclera (ppScl), leading to primary damage to the optic nerve axons [37,48].

The lamina cribrosa is a spacious fibrous network that provides structural support and nutritional supply (via capillaries) to the RGCs. It accounts for only one-third of the scleral thickness (ppScl), and its supporting elements constitute only 40% of the tissue volume. This means it must prevent deformation caused by IOP while also providing free space for the optic nerve axons. As a result, the LC is very weak and sensitive to biomechanical and ischemic damage. High stresses caused by IOP can narrow the lumen of the lamina’s fenestrations and restrict blood flow [49].

The biomechanics of the eye constantly change and adapt to alterations in the eyeball. With age, the lamina cribrosa undergoes stiffening and sclerosis [50]. Remodeling of the lamina cribrosa occurs primarily because of increased intraocular pressure, hypoxia, oxidative stress, or even changes in cerebrospinal fluid pressure. Primarily, the LC undergoes deformation and posterior displacement [48,49,51]. Wang et al. (2018) [52] reported increased length and tortuosity of the lamina cribrosa pores in patients with glaucoma, which may also contribute to greater mechanical stress on the optic nerve axons. These changes exacerbate hypoperfusion and ischemia of the ppScl and optic nerve axons. Elevated TGF-β levels have also been reported, suggesting lamina cribrosa fibrosis during glaucoma progression [48,49,51].

Girkin et al. [53] conducted a study analyzing changes in lamina cribrosa (LC) depth and curvature in patients with primary open-angle glaucoma (POAG). In addition, the authors compared the progression of biomechanical changes in the LC with visual field progression. OCT scans were performed on 24 eyes. The authors demonstrated that, as glaucoma progressed, progressive posterior thinning of the LC (*p* < 0.001) and increased LC curvature (*p* < 0.001) were observed. Furthermore, faster progression of visual field defects was associated with these changes. This association was weaker in older individuals. Older patients had less LC remodeling despite the functional progression of glaucoma. Furthermore, patients with higher intraocular pressure (*p* = 0.030) and thinner central corneal thickness (*p* < 0.001) exhibited greater posterior displacement of the LC.

**Figure 2:**
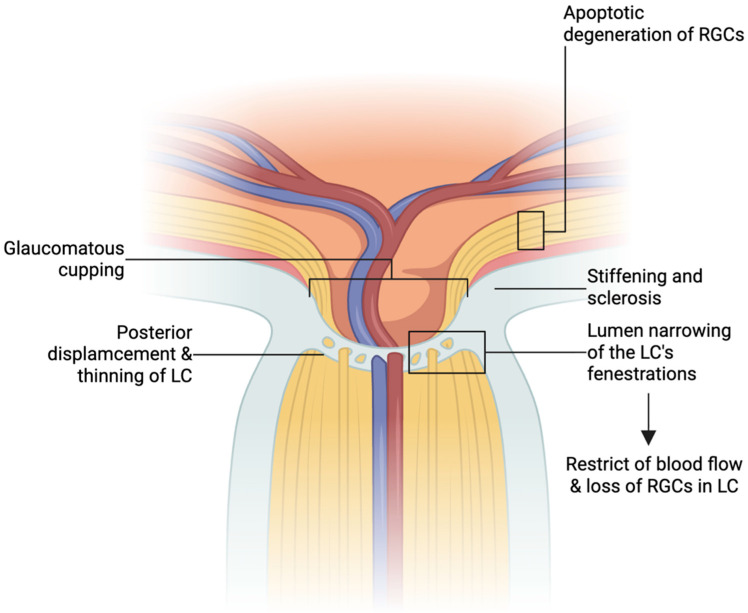
Major biomechanical changes in glaucomatous optic nerve head. *RGCs—retinal ganglion cells; LC—lamina cribrosa.* Created in BioRender. Mazurek, W. (2026) https://BioRender.com/llzltot (accessed on 25 May 2026).

Mechanical damage to the lamina cribrosa may result from increased IOP, and disturbances in nutrient supply are interrelated. The vessels supplying the LC run through the supporting tissues, which are subject to stress and deformation. Thus, changes in the biomechanics of the lamina cribrosa can simultaneously alter the shape, stiffness, and microenvironment of the vessels, affecting flow and diffusion. This may compromise axonal metabolic support, ultimately promoting retinal ganglion cell degeneration [49]. Considering biomechanical parameters can contribute to a more accurate glaucoma diagnosis and provide a better understanding of the mechanism of optic nerve damage [54]. Figure 2 shows the mentioned changes in the optic disc in glaucoma.

### 2.3. Hypoxia and Oxidative Stress in Glaucomatous Neurodegeneration

Optic nerve hypoxia is primarily caused by decreased blood flow (hypoperfusion) [25,55]. In response to inadequate oxygen availability, transcription factors known as hypoxia-inducible factors (HIF) are expressed. Hypoxia inhibits HIF hydroxylation, leading to the accumulation of HIF-1α, which in turn triggers a cascade of gene transcription involved in erythropoiesis, angiogenesis, and energy metabolism—the hypoxia response mechanism [56]. Thus, HIF-1α is a marker of tissue hypoxia.

Feng et al. [57] investigated the role of lamina cribrosa astrocytes in the autoregulation of blood flow in the optic nerve head. The authors used the oxygen-glucose deprivation/reperfusion (OGD/R) model in an in vitro study. They showed that hypoxia inhibits the proliferation of lamina cribrosa astrocytes and induces their apoptosis. After reoxygenation, there was upregulation of glial fibrillary acidic protein (GFAP), mechanistic target of rapamycin (mTOR), and cytosolic phospholipase A_2_ (cPLA2), as well as an increase in the concentration of prostaglandin E2 (PGE2). This led to secondary vasodilation. This provides evidence that astrocytes may contribute to autoregulatory mechanisms in the ONH.

Further evidence comes from animal models. Chidlow et al. [58] conducted an experimental study using a rat glaucoma model in which IOP was unilaterally increased by injecting microparticles into the anterior chamber, with the contralateral eye serving as a control. Pimonidazole staining was used to detect hypoxia. They also analyzed markers of oxidative stress and assessed axonal transport using anterograde tracers. The authors demonstrated that induction of elevated IOP increases hypoxia signaling in the ONH, particularly at the lamina cribrosa (*p* < 0.05). Areas of hypoxia correlated with sites of impaired axonal transport. An increase in oxidative stress markers (HO-1, Cfos, p-cJun) was observed compared to the control (*p* < 0.05). A statistically significant association was demonstrated between hypoxia, oxidative stress, and axonal dysfunction in the studied model of high-pressure glaucoma.

In the study conducted by Jassim et al. [59], a transgenic mouse model was used to map IOP-induced hypoxia throughout the visual pathway. The authors investigated the topography of HIF1α-dependent hypoxia. Results showed that elevated IOP leads to rapid (3 h to 3 days) and widespread hypoxia not only in the retina and optic nerve but also in the visual centers of the brain (superior colliculus and lateral geniculate nucleus). Hypoxia primarily affected Müllerian cells, astrocytes, and microglia in the optic nerve. The highest severity of hypoxia was observed in the early stages following IOP induction. Furthermore, increased expression of glucose transporters (GLUT1 and GLUT3) was observed, indicating metabolic adaptation of tissues to oxygen deprivation. These results suggest that hypoxia is an early and persistent pathophysiological mechanism in high-pressure glaucoma, affecting the entire visual system and not just the eyeball.

Another study performed by Jassim et al. [60], the DBA/2F (D2) mouse model of chronic glaucoma, and the DBA/2J-Gpnmb (D2G) control group was used to analyze retinal and optic nerve changes at multiple time points. In the D2 group, a significant increase in IOP and progressive loss of RGCs and axons were observed (*p* < 0.05–0.0001). An increase in ROS and a decrease in SOD2 activity were noted, indicating mitochondrial dysfunction. Additionally, hypoxia in RGCs and a significant increase in HIF-1α in later stages of the disease were demonstrated (*p* = 0.0074). This paper points to the multifactorial pathogenesis of glaucoma. It can be linked to the mechanical hypothesis (connected with IOP), but importantly to theories involving hypoxia, oxidative stress, and mitochondrial dysfunction.

Human studies have extended the above observations, providing direct evidence of hypoxia and impaired oxygenation in glaucomatous optic neuropathy. Tezel and Wax [61] investigated retinal and ONH hypoxia in glaucomatous eyes by analyzing HIF-1α. They examined 28 human glaucoma donors compared with 20 control eyes from healthy donors. The authors found increased HIF-1α levels in the retina and optic nerve head in the study group. This study demonstrated that hypoxia is a likely component of the pathogenesis of neurodegeneration in glaucoma. A limitation of the study was the postmortem tissue analysis. Furthermore, while the increased expression of HIF-1α was correctly associated with hypoxia, this does not directly prove the mechanism of the damage.

Reszeć et al. [62] examined HIF-1 expression in RGCs and optic nerve axons in advanced glaucoma. The study included 42 eyes removed due to advanced glaucoma and 2 control eyes removed after trauma. Immunohistochemistry with anti-HIF-1alpha antibody revealed HIF-1 expression in 57.1% of optic nerve axons and 52.3% of RGCs; no expression was observed in controls. The limitations of this study included the use of samples from removed eyes and the size of the control group. This study is rather similar to the work of Tezel and Wax [61]. Due to the fact that the tissues came from eyeballs harvested from donors, it is complicated to directly link these findings to glaucomatous neuropathy.

A particularly interesting study is the work conducted by Al Zoubi et al. [63] evaluated perfusion and hemoglobin oxygenation within the optic disc. The study group comprised patients with primary open-angle glaucoma (n = 31), while the control group consisted of a healthy cohort (n = 31). The authors showed that hemoglobin concentration was significantly lower in the rim of the control group (*p* < 0.001) and in the excavation (*p* < 0.001). In 58% of patients with glaucoma, oxygen saturation was significantly lower than in the remaining patients (*p* = 0.02) and the control group (*p* = 0.01). Hemoglobin concentration correlated with retinal nerve fiber layer (RNFL) thickness, peripapillary perfusion, and visual field defects, suggesting a link between perfusion disorders, hypoxemia, and disease severity.

The studies discussed above indicate that hypoxia and oxidative stress play a key role in glaucomatous neurodegeneration. They contribute to optic nerve damage regardless of whether IOP is elevated. The proposed mechanism involves reduced blood flow to the optic nerve, structural remodeling of the lamina cribrosa, and progressive apoptosis of RGCs. Notably, the same mechanisms are also activated in OSA, which may explain the causal link between the two conditions, which is discussed in the following section.

## 3. Pathophysiology of Obstructive Sleep Apnea

OSA results from repeated collapse of the upper airway [8]. The pharynx lacks skeletal support, and its patency depends on the active tone of the dilator muscles, which is reduced during sleep. As a result, the airway is prone to collapse, especially in patients with obesity, craniofacial abnormalities, or impaired muscle function [8,64]. Beyond these anatomical predispositions, the principal risk factors for OSA include obesity, male sex, and advancing age [7,8]. OSA is also associated with systemic hypertension: recurrent apneic episodes and the accompanying surges in sympathetic activity promote sustained elevations in blood pressure. In turn, OSA is highly prevalent among patients with resistant hypertension [65,66]. Because ocular perfusion pressure depends directly on systemic blood pressure, these hemodynamic disturbances are mechanistically relevant to the optic nerve perfusion.

Each obstructive event reduces or stops airflow. This causes a decrease in oxyhemoglobin saturation, a rise in arterial carbon dioxide, and an increase in negative intrathoracic pressure. A brief arousal usually terminates the episode. The resulting pattern consists of repetitive cycles of hypoxia and reoxygenation, accompanied by sleep fragmentation [8,67].

One of the main polysomnographic parameters in diagnosing OSA is the apnea–hypopnea index (AHI). The AHI represents the total number of apneas and hypopneas per hour during sleep. They are defined as incidents lasting at least 10 s and causing desaturation. The severity of OSA is classified according to the AHI into mild (5–15/h), moderate (15–30/h), and severe (>30/h) [68,69].

The key consequence of these events is chronic intermittent hypoxia (CIH). Cyclic desaturation activates carotid body chemoreceptors, leading to sustained sympathetic activity and the generation of reactive oxygen species [70,71]. These promote systemic oxidative stress and low-grade inflammation [67,72]. These mechanisms impair vascular endothelial function. The downstream molecular and vascular changes relevant to ocular perfusion are discussed below.

### Vascular and Molecular Dysregulation in Obstructive Sleep Apnea

Vascular risk factors, including migraine, Raynaud’s syndrome, diabetic microangiopathy [30], atrial fibrillation, and other conditions that reduce ocular perfusion, may contribute to the development of glaucomatous optic neuropathy. Importantly, disorders such as OSA can also result in hypoperfusion and hypoxia of the optic nerve head [10,12].

In OSA, blood vessel autoregulation is disrupted. This causes reduced vasodilation, increased vasospasm, inflammation, and coagulation. As a result, the vascular system loses elasticity, has impaired endothelial regeneration, and has diminished vascular reactivity. These changes are linked to reduced nitric oxide (NO) availability. Furthermore, chronic intermittent hypoxia is the main cause of these alterations [73].

Recurrent apneic episodes also cause significant nocturnal fluctuations in systemic blood pressure and activation of the sympathetic nervous system [74,75]. These nocturnal hemodynamic disturbances may further impair OPP and contribute to optic nerve ischemia, particularly in patients with normal-tension glaucoma, in whom vascular dysregulation is believed to play a key role in the pathogenesis of the disease [25].

In addition, CIH and recurrent reperfusion induce oxidative stress. This leads to an increase in reactive oxygen species (ROS) and cytokines (such as IL-6, TNF-α, IL-1β, CRP, ICAM-1, VCAM-1) [33,35,76]. It also contributes to decreased NO (a vasodilator) and elevated levels of endothelin-1 (ET-1) (a vasoconstrictor), exacerbating chronic microcirculatory damage and impaired perfusion [33,34]. Consequently, disorders of microcirculation and vascularization in the ONH can cause progressive damage to the nerve and are a risk factor for glaucoma [12]. Similar changes in cytokine expression may occur in patients with normal-tension glaucoma. According to Galassi et al. [77], nitric oxide levels were significantly lower in patients with NTG than in healthy individuals (*p* < 0.001), while ET-1 levels were significantly higher (*p* < 0.001).

Moreover, CIH causes damage to mitochondria, where hypoxia leads to excessive production of reactive oxygen species (ROS). These exacerbate mitochondrial damage and trigger a vicious cycle of oxidative stress. The authors also describe changes in the expression of genes regulating mitochondrial biogenesis (including PGC-1α and NRF1). Furthermore, they note disturbances in mitochondrial DNA homeostasis [78,79,80]. This is significant because mitochondrial dysfunction can be linked to one of the theories of glaucoma pathogenesis.

RGCs are particularly sensitive to mitochondrial damage and ATP deficiency. Mitochondrial dysfunction leads to the activation of inflammatory responses and oxidative stress, which causes damage to lipids, proteins, and DNA. Furthermore, ROS impairs glutamate metabolism, exacerbating mitochondrial damage and promoting apoptosis in retinal ganglion cells [24].

The factors causing glaucoma in OSA include a correlation between hypoperfusion (reduced OPP) and hypoxia. Optic nerve hypoxia is likely the result of multiple factors that form a vicious cycle in the pathogenesis. This includes vascular endothelial dysfunction, impaired vasodilation, increased vasoconstriction, oxidative stress, mitochondrial disturbance, and remodeling of the lamina cribrosa, which leads to progressive optic nerve ischemia (Figure 1) [24,27,37].

These mechanisms operate in a multifactorial manner. Repetitive hypoxia–reoxygenation (CIH) cycles promote sympathetic overactivity, endothelial dysfunction, and oxidative stress. As a result, OPP is reduced, destabilizing blood flow within the ONH. Hypoxia of the ONH leads to LC remodeling, which compresses the traversing capillaries and further reduces axonal nutrient supply. Oxidative stress is involved at every node of this circuit, acting both as a product of CIH and as a factor of vascular and mitochondrial injury. In this way, an initial perfusion deficit is gradually converted into progressive, IOP-independent retinal ganglion cell loss (Figure 1).

The mechanisms described above suggest a pathophysiologically plausible link between OSA and GON. Consequently, many authors have investigated the impact of obstructive sleep apnea on glaucoma, its prevalence, and the potential significance of the correlation between these conditions.

## 4. OSA and Glaucoma: Epidemiological Evidence

The literature describes a significant association between OSA and glaucoma. Nevertheless, much of the data is inconsistent or contradictory. Notably, some authors highlight the particular significance of OSA in patients with normal-tension glaucoma [5,12]. Nevertheless, the strength of this association appears to vary across study designs and patient characteristics. Table 1 summarizes the epidemiological evidence (Table 1). While Table 1 provides an at-a-glance summary of each study, the following subsections complement it by focusing on the methodological context, diagnostic criteria, and limitations needed to weigh these findings.

### 4.1. Prevalence of Glaucoma in Patients with OSA

Bendel et al. [81] conducted a study involving 100 patients with moderate-to-severe OSA. The patients underwent a 48 h polysomnographic examination. The patients also underwent several ophthalmological examinations. One of the main outcomes of the study was to determine the prevalence of glaucoma in patients with OSA. The prevalence of glaucoma among patients with OSA was estimated at 27%. Available studies consistently suggest a higher prevalence of glaucoma among patients with moderate-to-severe OSA, although the reported estimates vary substantially between cohorts and study designs.

Hashim et al. [82] performed a study that included 39 patients diagnosed with moderate OSA (n = 12) and severe OSA (n = 17). The authors showed that 20.5% of patients (n = 8) in the entire group were diagnosed with glaucoma. Analysis revealed that a higher prevalence of glaucoma was observed in patients with severe OSA. However, the authors did not prove statistical significance in these results.

In another study, Bagabas et al. [83] included 84 adult patients in their analysis. The authors showed that the prevalence of glaucoma was 16% among patients with OSA and 8% in patients without OSA. Although glaucoma was more common in the first group, this difference was not statistically significant.

Lin et al. [84] conducted a study comparing patients with OSA (n = 209) to healthy controls (n = 38). All individuals underwent an ophthalmological examination to diagnose normal-tension glaucoma (NTG). NTG was diagnosed in 5.7% of patients (n = 12) with obstructive sleep apnea, which was significantly more common than in the control group (n = 0) (*p* = 0.003). Furthermore, the authors demonstrated that NTG prevalence was higher in severe OSA (*p* = 0.033).

The studies above suggest a higher prevalence of glaucoma in patients with OSA, with reported rates ranging from 5.7% to 27% [81,82,83,84]. The strongest evidence comes from Lin et al., who demonstrated a significantly higher prevalence of NTG in OSA patients compared with healthy controls (*p* = 0.003) [84]. Several studies also indicate that the association is more pronounced in patients with severe OSA. However, these findings should be interpreted with caution. The cohorts were small, two studies lacked a control group, and the diagnostic criteria for both glaucoma and OSA varied across studies. Despite these limitations, the available evidence consistently suggests a potential link between OSA and glaucoma, particularly NTG, warranting further investigation in larger prospective studies.

### 4.2. Prevalence of OSA in Patients with Glaucoma

One of the most relevant meta-analyses was conducted by Yu et al., who assessed the prevalence of OSA in patients with glaucoma. A total of 544 publications and 40 additional records were identified, from which 956 patients were ultimately included in the analysis. The authors found that 17% of patients with OSA had glaucoma [85].

A similar trend was observed by Bilgin et al. [86], who compared 24 patients with NTG to 24 controls without glaucoma (with similar risk factors, such as diabetes mellitus, hypertension, and hypercholesterolemia). The patients underwent polysomnography and AHI assessment. Among patients with NTG, obstructive sleep apnea was present in 41.7% of individuals, compared to 12.5% in the control group. This difference was statistically significant (*p* < 0.05).

### 4.3. The Association Between Obstructive Sleep Apnea and Glaucoma

In a retrospective case–control study conducted by Funk et al. [6] at the Mayo Clinic in Rochester, the authors identified risk factors for low-tension glaucoma (LTG; classified as NTG). It was demonstrated that patients with LTG showed a strong association with hypertension (*p* = 0.004), diabetes mellitus (*p* < 0.001), peripheral vascular disease (*p* = 0.009), migraine (*p* = 0.02), anemia (*p* = 0.003), systemic hypotension (*p* < 0.001), and Raynaud’s syndrome (*p* = 0.05). However, no statistically significant differences were observed between the groups in the prevalence of obstructive sleep apnea.

In contrast, a meta-analysis conducted by Cheong et al. [87], involving 46 studies and over 4.5 million patients, showed that OSA is significantly associated with a high risk of glaucoma (*p* < 0.01). Taking other risk factors into account, it was found that patients with OSA have up to a 40% higher risk of developing glaucomatous neuropathy. Furthermore, Garcia-Sanchez et al. [88] in another meta-analysis showed that obstructive sleep apnea significantly increases the risk of glaucoma (*p* < 0.001), nonarteritic anterior ischemic optic neuropathy (NAION) (*p* < 0.001), and diabetic retinopathy (*p* = 0.02). The authors presented pathogenic pathways similar to those described in our article. In 2015, Shi et al. [89] presented a meta-analysis and literature review that included 16 studies involving over 2 million patients (6 case–control and 9 cross-sectional studies). The meta-analysis revealed a statistically significant association between OSA and glaucoma incidence, confirming previous data. In another meta-analysis, Liu et al. [90] included 6 studies (3 case–control and 3 cohort) and encompassed over 2 million patients. The authors demonstrated a significant association between OSA and glaucoma. For the case–control studies, the adjusted odds ratio was OR = 2.46 (*p* = 0.005). On the other hand, for the cohort studies, the OR was 1.43 (*p* < 0.001).

Importantly, Huon et al. [91] examined the association between obstructive sleep apnea and ophthalmic conditions, including glaucoma, NAION, retinal vein occlusion (RVO), central serous chorioretinopathy (CSR), and floppy eyelid syndrome (FES). A statistically significant correlation (*p* < 0.05) was found in all the mentioned conditions. The authors emphasized the importance of ophthalmic evaluation in OSA.

Zoh et al. [92] conducted a cross-sectional study involving over 9,000 individuals. They demonstrated a statistically significant association between OSA and glaucoma (*p* = 0.044). However, the main limitation of this study was the absence of polysomnography or polygraphy, which remain the gold standard for the diagnosis of OSA. Instead, the risk of OSA was assessed using the STOP-BANG questionnaire.

## 5. Effects of Obstructive Sleep Apnea on Glaucoma Factors

Many general factors can increase the risk of developing glaucoma or related eye conditions. Theoretically, many systemic diseases lead to microcirculatory dysfunction, which has a proven effect on glaucoma or a presumed potential effect on its progression [6,93]. Similarly, obstructive sleep apnea is actually a factor that may contribute to the development of glaucoma. In some cases, this leads to nerve damage before full-blown glaucoma develops [12]. Therefore, it is difficult to discuss the direct impact of OSA on glaucoma. In this study, we analyze which factors may influence the development of GON in patients with OSA.

### 5.1. Retinal Nerve Fiber Layer (RNFL)

In general, most studies report a significant difference in retinal nerve fiber layer (RNFL) thickness in OSA. The degree of RNFL thinning is often associated with the stage of OSA. However, authors do not fully agree on which ONH quadrants are affected [94,95,96,97,98,99,100]. Taken together, these studies indicate that RNFL thinning may represent one of the earliest structural manifestations of optic nerve vulnerability in OSA.

Zhao et al. [101] included six articles in their meta-analysis. The study encompassed 1034 eyes. The authors found a significant overall reduction in RNFL thickness in patients with OSA compared to healthy controls (*p* = 0.01). The RNFL thinning increased with OSA severity. Interestingly, the authors demonstrated significantly more frequent thinning in the lower quadrant of the optic disc (*p* = 0.02). In another meta-analysis, Wang et al. (2017) [102] demonstrated a significant reduction in RNFL in all quadrants of the optic nerve (*p* < 0.001 for superior and inferior, *p* = 0.003 for nasal, and *p* = 0.025 for temporal). As in a previous study, a significant reduction in RNFL was observed in correlation with the severity of obstructive sleep apnea.

An important cross-sectional study was conducted by Kargi et al. [103], who included 66 patients admitted for polysomnography. The study group consisted of patients with OSA (mild and severe), while healthy patients were assigned to the control group. Prior to analysis, 66 participants with glaucoma and other eye diseases that could affect the RNFL were excluded. Consequently, the final analysis included 34 patients with OSA and 15 controls. A statistically significant reduction in RNFL thickness was demonstrated in patients with OSA compared to controls. Furthermore, RNFL thinning correlated with OSA severity (*p* = 0.01). These findings suggest that changes during OSA, including hypoxia and decreased OPP, may lead to RNFL thinning. Compared to the previous study, the prospective trial conducted by Teberik et al. [104] found no difference in RNFL thickness (*p* = 0.274) between the OSA group (n = 103) and healthy controls (n = 37). Interestingly, the authors found lower IOP and CCT values in patients with OSA, but this difference was not statistically significant. Thus, this study did not reveal any differences in changes in RNFL, IOP, or CCT. A limitation of the study was the significantly smaller control group than in the study by Kargi et al. [103].

A retrospective cohort study by Fan et al. [105] demonstrated a higher rate of RNFL thickness reduction with increasing OSA severity (*p* = 0.042). After adjusting for demographic factors (age, sex, diabetes, hypertension, hyperlipidemia, and body mass index), severe OSA was associated with an over 8-times increased risk of RNFL thickness reduction (*p* = 0.017).

In a prospective cross-sectional study conducted by Devi et al. [106], 90 patients (90 eyes) with diagnosed OSA were analyzed. Patients were stratified into three OSA severity levels based on their apnea–hypopnea index (AHI). A statistically significant decrease in RNFL thickness was observed as OSA severity increased (*p* < 0.05). Interestingly, the upper quadrant of the RNFL was significantly reduced in all groups.

Ngoo et al. [107] compared 54 patients with OSA and 54 healthy controls. In the study group, the mean RNFL thickness was estimated at 93.87 µm (*p* < 0.05). The upper quartile was 113.59 µm (*p* ≤ 0.001). In contrast, in the control group, the mean RNFL thickness was 98.96 µm (*p* < 0.05), while the upper quartile was 125.76 µm (*p* < 0.05). A significant difference in RNFL thickness between groups was demonstrated, and a correlation between RNFL reduction and AHI severity was observed.

The summary of the articles presented above is provided in Table 2.

### 5.2. Cup-to-Disc Ratio (CDR)

In the analyzed articles, most authors agree that there is no significant increase in the cup-to-disc ratio in individuals with OSA [87,107,108,109,110]. CDR primarily reflects gross structural changes in the ONH, which might not manifest in early or subclinical stages of optic nerve damage, whereas RNFL thinning can detect more subtle, preperimetric alterations.

### 5.3. Intraocular Pressure

Ferrandez et al. [97] conducted a prospective study involving 40 patients with OSA and 45 healthy controls. In the study group, patients were stratified into three groups based on the AHI. The authors demonstrated that IOP averaged 14.23 ± 2.6 mmHg in OSA patients, whereas in the control group, it was significantly higher at 17.42 ± 2.6 mmHg (*p* < 0.001). This finding ran counter to the expected direction.

Moghimi et al. [111] involved 51 patients with OSA. The study group was divided into three strata based on the AHI. The authors demonstrated that 6.7% had an IOP >21 mmHg, which was statistically significant (*p* < 0.001, even after adjustment for CCT). In a study by Çekiç et al. [112], the authors compared 30 patients with severe OSA (AHI > 30) with a control group of 28 healthy patients. As in the previous paper, this study demonstrated that patients with severe OSA had significantly higher IOP than the control group (*p* < 0.05).

Nevertheless, a significant number of authors report no correlation between IOP and obstructive sleep apnea [113,114,115,116,117,118,119]. Asker et al. [120] studied 41 patients with OSA and 17 controls. The authors reported a mean IOP of 15.5 ± 3.0 mmHg in the OSA group and 13.5 ± 1.7 mmHg in the control group. No significant difference was found between the groups (*p* = 0.1). Chirapapaisan et al. [109] analyzed 41 patients with OSA, finding that IOP was elevated in the severe OSA group but remained within the reference range.

In a prospective observational study conducted by Carnero et al. [121], intraocular pressure fluctuations were assessed during the day in patients with OSA. The authors used a contact lens sensor (CLS) to monitor IOP in 20 patients with OSA. It was shown that patients with OSA exhibited significant nocturnal fluctuations in IOP, which occurred mainly during apneic episodes and periods of decreased oxygen saturation. However, a limitation of the study was the absence of a control group.

The data in the literature are inconsistent and contradictory. Some studies show significantly elevated IOP in patients with OSA [111,112], while others find no statistical significance [113,114,115,116,117,118,119,120]. This inconsistency may partly result from differences in patient selection, OSA severity, and methods of IOP assessment. Overall, current findings suggest that OSA may influence IOP regulation predominantly through nocturnal fluctuations and perfusion instability rather than through sustained daytime IOP elevation alone.

### 5.4. Visual Field (VF)

The visual field (VF) plays a key role in the diagnosis and monitoring of glaucoma and other optic neuropathies. The 24-2 visual field test with Swedish Interactive Thresholding Algorithm (SITA) is currently recommended for assessing the visual field in GON [122,123]. In the clinic, mean deviation (MD) is used to stratify glaucoma, enabling the assessment of visual field deterioration and monitoring in patients. MD is the average of all values on the VF and is typically negative. Visual field defects result in a higher deviation [124]. In the context of OSA, functional visual field alterations appear less consistent than structural optic nerve changes, which may reflect the subclinical nature of early glaucomatous damage in these patients.

A meta-analysis conducted by Cheong et al. [87] demonstrated a statistically significant difference in the VF defects among patients with OSA (*p* = 0.02). However, some studies have reported contradictory results.

Davanian et al. [125] found that obstructive sleep apnea did not have a statistically significant effect on the deterioration of visual field parameters. In addition, Salzgeber et al. [126] reached similar conclusions, demonstrating that the VF of patients with OSA did not differ from that of the healthy control group.

In contrast, Sergi et al. [127] demonstrated that the severity of OSA correlates with the deterioration of visual field parameters (*p* < 0.05). Similarly, Casas et al. [128] showed that the mean MD in the OSA group was −0.64 ± 1.38 (−4.87 to 1.65), whereas that in the healthy control group was 0.07 ± 1.01 (−2.78 to 1.79). The difference was statistically significant (*p* = 0.002).

In patients with OSA, changes in the VF are less consistent than structural alterations in the RNFL. This may be due to preperimetric RNFL damage. The cited papers report contradictory results. Some studies show a significant association between VF sensitivity and the severity of OSA [87,127,128]. Nevertheless, studies conducted by Davanian et al. [125] and Salzgeber et al. [126] found no significant differences. The conflicting results may stem from differences in OSA severity, patient selection, and the sensitivity of standard perimetry in detecting early subclinical changes.

## 6. The Effect of CPAP Treatment on Development of Glaucoma

Continuous positive airway pressure (CPAP) is the first-line treatment for moderate-to-severe OSA. It maintains upper airway patency and prevents recurrent hypoxia during sleep. The effect of CPAP on IOP remains a subject of debate, with some studies reporting an increase in IOP, others showing no change, and a few demonstrating beneficial effects on the IOP profile [129,130].

One of the earlier studies was conducted by Kiekens et al. [131], who measured IOP every 2 h during 24 h sessions in 21 patients with newly diagnosed OSA. The authors demonstrated a significant increase in the average IOP after one month of CPAP therapy. A similar effect was reported by Hirunpatravong et al. [132], who examined 12 eyes of patients with POAG and OSA for 12 months. The mean IOP increased from 17.83 to 19.08 mmHg after CPAP treatment (*p* < 0.05). Importantly, no progression of glaucomatous damage was observed during follow-up, as visual field indices remained stable.

In contrast, Pepin et al. [133] reported a different effect of CPAP on the nyctohemeral rhythm of IOP. The authors analyzed 24 h IOP and ocular perfusion pressure in 18 patients with severe OSA, of whom 12 were reassessed after CPAP treatment. They demonstrated that the normal and physiological nyctohemeral IOP rhythm was lost in most patients with severe apnea. Treatment with CPAP restored a normal 24 h IOP profile in most cases. Furthermore, Himori et al. [134] showed that CPAP therapy in patients with both glaucoma and OSA reduced systemic oxidative stress and improved the visual field. The authors suggested that CPAP may have a neuroprotective effect that extends beyond its direct effects on IOP.

These inconsistent findings have been addressed in several recent meta-analyses. Kongchan et al. [130] analyzed 15 studies involving 495 participants and reported no significant difference in the pooled mean IOP after CPAP therapy compared with baseline. However, subgroup analysis revealed significantly higher IOP after in-laboratory titration and after long-term CPAP use. On the other hand, Chan et al. [135] reported that short-term CPAP of one month or less was associated with elevated IOP, while long-term effects were minimal. The authors emphasized that caution is warranted when initiating CPAP in patients with established glaucoma.

The available evidence suggests that CPAP may transiently raise IOP, particularly during the early phase of therapy. However, long-term effects appear minimal, and CPAP may even restore normal IOP dynamics and reduce oxidative stress in selected patients. These effects probably stem from different mechanisms. In the first weeks of treatment, the positive airway pressure tends to raise IOP. Over longer periods, the attenuation of intermittent hypoxia and oxidative stress seems to offset that early increase. Determining which effect dominates will require prospective studies that stratify patients by glaucoma type and baseline IOP, and that track both structural (RNFL) and functional (visual field) outcomes over a longer observation period. Despite these findings, the quality of evidence remains limited and largely based on small observational studies. Well-designed randomized controlled trials with longer follow-up are needed to confirm whether CPAP affects the progression of glaucoma in patients with OSA.

## 7. Conclusions

Obstructive sleep apnea and glaucoma share several pathophysiological mechanisms, the most important of which appears to be CIH. By lowering OPP and promoting lamina cribrosa remodeling and oxidative stress, CIH can damage retinal ganglion cells largely independently of IOP, which may explain why NTG is the form most often associated with OSA. The reported strength of this association varies across studies, mainly because of differences in study design, OSA severity grading, and glaucoma criteria. Prospective studies with standardized endpoints and randomized trials of CPAP are still needed. In practice, patients with OSA may benefit from ophthalmological assessment for early IOP-independent optic nerve damage.

## Figures and Tables

**Table 1:** Summary of the epidemiological evidence: NTG—normal-tension glaucoma; OSA—obstructive sleep apnea.

Study (Year)	Article Type	n	Glaucoma Type	Key Finding	Statistical Significance	Limitations
**Prevalence of Glaucoma in Patients with OSA**						
Bendel et al. (2008) [81]	Cross-sectional	100	Any type	27% prevalence of glaucoma in OSA patients	Not reported	No control group, no mild OSA included
Hashim et al. (2014) [82]	Prospective cohort	39	Any type	20.5% diagnosed with glaucoma, higher in severe OSA	Not significant	Small sample, no control group, no mild OSA included
Bagabas et al. (2019) [83]	Cross-sectional	84	Any type	16% prevalence of glaucoma in OSA group vs 8% in non-OSA group	Not significant	Variable diagnostic criteria
Lin et al. [84]	Cross-sectional	247 (OSA 209, controls 38)	NTG	NTG 5.7% in patients with OSA vs. 0% in controls	*p* = 0.003	Small control group
**Prevalence of OSA in patients with glaucoma**						
Yu et al. (2021) [85]	Systematic review and meta-analysis	956	Any type	17% of glaucoma patients was diagnosed with OSA	Significant	Study heterogeneity, small group of studies, small effect sizes
Bilgin et al. (2014) [86]	Prospective case–control	48 (NTG 24, controls 24)	NTG	OSA in 41.7% of NTG vs. 12.5% in controls	*p* < 0.05	Small samples, single center
**The association between OSA and glaucoma**						
Funk et al. (2022) [6]	Retrospective case–control	554 (277 patients in study and control groups)	NTG (low-tension)	No significant association	Not significant	Retrospective, single center
Cheong et al. (2023) [87]	Meta-analysis	>4.5 million (46 studies)	Any type	OSA associated with up to 40% higher risk of glaucoma	*p* < 0.01	Heterogeneity
Garcia-Sanchez et al. (2022) [88]	Meta-analysis	107 studies	Any type	OSA associated with 50% higher risk of glaucoma	*p* < 0.001	Heterogeneity
Shi et al. (2015) [89]	Meta-analysis	>2 million (6 studies)	Any type	Significant association	Significant	Heterogeneity
Liu et al. (2016) [90]	Meta-analysis	>2 million (6 studies)	Any type	Significant association	Case–control studies *p* = 0.005; cohort studies *p* < 0.001	Few studies
Huon et al. (2016) [91]	Meta-analysis	31 studies	Any type	Significant association	*p* < 0.05	
Zoh et al. (2025) [92]	Cross-sectional	>9000	Any type	Significant association	*p* = 0.044	No polysomnography, questionnaire-based OSA diagnosis

**Table 2:** Summary of RNFL changes in patients with OSA: NTG—normal-tension glaucoma; OSA—obstructive sleep apnea; OCT—optical coherence tomography; RNFL—Retinal Nerve Fiber Layer.

Study (Year)	Article Type	n	Method	RNFL Findings	Quadrants	Correlation with AHI	Limitations
**Meta-analyses**							
Zhao et al. (2016) [101]	Meta-analysis	1034 eyes (6 studies)	OCT	Overall RNFL reduction	Inferior (*p* = 0.02)	Yes	Heterogeneity, only 6 studies
Wang et al. (2017) [102]	Meta-analysis	1757 eyes (1106 in OSA group and 651 controls)	OCT	Significant reduction	All quadrants (superior and inferior *p* < 0.001, nasal *p* = 0.003, temporal *p* = 0.025)	Yes	Heterogeneity
**Individual studies with RNFL reduced**							
Kargi et al. (2005) [103]	Cross-sectional	34 OSA, 15 controls	OCT	Significant reduction	All quadrants	*p* = 0.01	
Fan et al. (2019) [105]	Retrospective cohort	32	OCT	Thickness reduction with OSA severity. Severe OSA 8x increased risk of RNFL thinning	Not specified	*p* = 0.042; severe OSA *p* = 0.017	Retrospective
Devi et al. (2023) [106]	Prospective cross-sectional	90	OCT	Significant RNFL decrease with OSA severity	Superior	*p* < 0.05	No control group
Ngoo et al. (2021) [107]	Cross-sectional	54 OSA, 54 controls	OCT	OSA: Mean RNFL 93.87 µm; Controls: 98.96 µm	Superior (*p* ≤ 0.001)	Yes	Single center
**Individual studies with RNFL not significantly reduced**							
Teberik et al. (2018) [104]	Prospective cross-sectional	103 OSA, 37 controls	OCT	No significant difference in RNFL; lower IOP and CCT in OSA	None	*p* = 0.274	Small control group; no severity stratification

## Data Availability

Data are contained within the article.

## References

[1] Jonas J.B., Aung T., Bourne R.R., Bron A.M., Ritch R., Panda-Jonas S. (2017). Glaucoma. Lancet.

[2] Michels T.C., Ivan O. (2023). Glaucoma: Diagnosis and Management. Am. Fam. Physician.

[3] Schuster A.K., Erb C., Hoffmann E.M., Dietlein T., Pfeiffer N. (2020). The Diagnosis and Treatment of Glaucoma. Dtsch. Arztebl. Int..

[4] Tham Y.C., Li X., Wong T.Y., Quigley H.A., Aung T., Cheng C.Y. (2014). Global prevalence of glaucoma and projections of glaucoma burden through 2040: A systematic review and meta-analysis. Ophthalmology.

[5] Dada T., Verma S., Gagrani M., Bhartiya S., Chauhan N., Satpute K., Sharma N. (2022). Ocular and Systemic Factors Associated with Glaucoma. J. Curr. Glaucoma Pract..

[6] Funk R.O., Hodge D.O., Kohli D., Roddy G.W. (2022). Multiple Systemic Vascular Risk Factors are Associated with Low-Tension Glaucoma. J. Glaucoma.

[7] Jordan A.S., McSharry D.G., Malhotra A. (2014). Adult obstructive sleep apnoea. Lancet.

[8] Lévy P., Kohler M., McNicholas W.T., Barbé F., McEvoy R.D., Somers V.K., Lavie L., Pépin J.-L. (2015). Obstructive sleep apnoea syndrome. Nat. Rev. Dis. Prim..

[9] Yeghiazarians Y., Jneid H., Tietjens J.R., Redline S., Brown D.L., El-Sherif N., Mehra R., Bozkurt B., Ndumele C.E., Somers V.K. (2021). Obstructive Sleep Apnea and Cardiovascular Disease: A Scientific Statement From the American Heart Association. Circulation.

[10] Faridi O., Park S.C., Liebmann J.M., Ritch R. (2012). Glaucoma and obstructive sleep apnoea syndrome. Clin. Exp. Ophthalmol..

[11] Leggewie B., Gouveris H., Bahr K. (2022). A Narrative Review of the Association Between Obstructive Sleep Apnea and Glaucoma in Adults. Int. J. Mol. Sci..

[12] Chaitanya A., Pai V.H., Mohapatra A.K., Ve R.S. (2016). Glaucoma and its association with obstructive sleep apnea: A narrative review. Oman J. Ophthalmol..

[13] Chowdhury U.R., Hann C.R., Stamer W.D., Fautsch M.P. (2015). Aqueous Humor Outflow: Dynamics and Disease. Investig. Ophthalmol. Vis. Sci..

[14] Killer H.E., Pircher A. (2018). Normal tension glaucoma: Review of current understanding and mechanisms of the pathogenesis. Eye.

[15] Alasbali T. (2023). Current State of Knowledge in Ocular Blood Flow in Glaucoma: A Narrative Review. Clin. Ophthalmol..

[16] Kim K.E., Park K.H. (2016). Update on the Prevalence, Etiology, Diagnosis, and Monitoring of Normal-Tension Glaucoma. Asia Pac. J. Ophthalmol..

[17] Qin Z., Meng L., Yang F., Zhang C., Wen B. (2021). Aqueous humor dynamics in human eye: A lattice Boltzmann study. Math. Biosci. Eng..

[18] Tanna A.P., American Academy of Ophthalmology (2021). Intraocular Pressure and Aqueous Humor Dynamics. Glaucoma, Basic and Clinical Science Course (BCSC), Section 10.

[19] Pizzirani S. (2015). Definition, Classification, and Pathophysiology of Canine Glaucoma. Vet. Clin. N. Am. Small Anim. Pract..

[20] Flammer J. (1994). The vascular concept of glaucoma. Surv. Ophthalmol..

[21] Feng K.M., Tsung T.H., Chen Y.H., Lu D.W. (2023). The Role of Retinal Ganglion Cell Structure and Function in Glaucoma. Cells.

[22] Mozaffarieh M., Grieshaber M.C., Flammer J. (2008). Oxygen and blood flow: Players in the pathogenesis of glaucoma. Mol. Vis..

[23] Zhang N., Wang J., Li Y., Jiang B. (2021). Prevalence of primary open angle glaucoma in the last 20 years: A meta-analysis and systematic review. Sci. Rep..

[24] Fernández-Albarral J.A., Ramírez A.I., de Hoz R., Matamoros J.A., Salobrar-García E., Elvira-Hurtado L., López-Cuenca I., Sánchez-Puebla L., Salazar J.J., Ramírez J.M. (2024). Glaucoma: From pathogenic mechanisms to retinal glial cell response to damage. Front. Cell. Neurosci..

[25] Flammer J., Orgül S., Costa V.P., Orzalesi N., Krieglstein G.K., Serra L.M., Renard J.-P., Stefánsson E. (2002). The impact of ocular blood flow in glaucoma. Prog. Retin Eye Res..

[26] Lee N.Y., Shin D.Y., Park C.K. (2024). Associations of long-term fluctuation in blood pressure and ocular perfusion pressure with visual field progression in normal-tension glaucoma. BMC Ophthalmol..

[27] Cherecheanu A.P., Garhofer G., Schmidl D., Werkmeister R., Schmetterer L. (2013). Ocular perfusion pressure and ocular blood flow in glaucoma. Curr. Opin. Pharmacol..

[28] Samsudin A., Isaacs N., Tai M.L.S., Ramli N., Mimiwati Z., Choo M.M. (2016). Ocular perfusion pressure and ophthalmic artery flow in patients with normal tension glaucoma. BMC Ophthalmol..

[29] He Z., Nguyen C.T.O., Armitage J.A., Vingrys A.J., Bui B.V. (2012). Blood Pressure Modifies Retinal Susceptibility to Intraocular Pressure Elevation. PLoS ONE.

[30] McMonnies C.W. (2016). Glaucoma history and risk factors. J. Optom..

[31] (2001). Collaborative Normal-Tension Glaucoma Study Group. Natural history of normal-tension glaucoma. Ophthalmology.

[32] Chan N.T.H., Pattamatta U., White A. (2025). Role of reactive oxygen species and oxidative stress in the pathomechanism of glaucoma. Med. Hypothesis Discov. Innov. Ophthalmol..

[33] Hoyos C.M., Melehan K.L., Liu P.Y., Grunstein R.R., Phillips C.L. (2015). Does obstructive sleep apnea cause endothelial dysfunction? A critical review of the literature. Sleep Med. Rev..

[34] Atkeson A., Jelic S. (2008). Mechanisms of endothelial dysfunction in obstructive sleep apnea. Vasc. Health Risk Manag..

[35] Peracaula M., Torres D., Poyatos P., Luque N., Rojas E., Obrador A., Orriols R., Tura-Ceide O. (2022). Endothelial Dysfunction and Cardiovascular Risk in Obstructive Sleep Apnea: A Review Article. Life.

[36] Patt B.T., Jarjoura D., Haddad D.N., Sen C.K., Roy S., Flavahan N.A., Khayat R.N. (2010). Endothelial dysfunction in the microcirculation of patients with obstructive sleep apnea. Am. J. Respir. Crit. Care Med..

[37] Grieshaber M.C., Flammer J. (2005). Blood flow in glaucoma. Curr. Opin. Ophthalmol..

[38] Izzotti A., Bagnis A., Saccà S.C. (2006). The role of oxidative stress in glaucoma. Mutat. Res. Rev. Mutat. Res..

[39] Stefánsson E., Pedersen D.B., Jensen P.K., La Cour M., Kiilgaard J.F., Bang K., Eysteinsson T. (2005). Optic nerve oxygenation. Prog. Retin Eye Res..

[40] Leske M.C., Wu S.Y., Hennis A., Honkanen R., Nemesure B. (2008). Risk Factors for Incident Open-Angle Glaucoma: The Barbados Eye Studies. Ophthalmology.

[41] Wang L., Cull G.A., Piper C., Burgoyne C.F., Fortune B. (2012). Anterior and posterior optic nerve head blood flow in nonhuman primate experimental glaucoma model measured by laser speckle imaging technique and microsphere method. Investig. Ophthalmol. Vis. Sci..

[42] Bonomi L., Marchini G., Marraffa M., Bernardi P., Morbio R., Varotto A. (2000). Vascular risk factors for primary open angle glaucoma: The Egna-Neumarkt Study. Ophthalmology.

[43] Topouzis F., Coleman A.L., Harris A., Jonescu-Cuypers C., Yu F., Mavroudis L., Anastasopoulos E., Pappas T., Koskosas A., Wilson M.R. (2006). Association of Blood Pressure Status with the Optic Disk Structure in Non-glaucoma Subjects: The Thessaloniki Eye Study. Am. J. Ophthalmol..

[44] Quigley H.A., West S.K., Rodriguez J., Munoz B., Klein R., Snyder R. (2001). The prevalence of glaucoma in a population-based study of Hispanic subjects: Proyecto VER. Arch. Ophthalmol..

[45] Weinreb R.N., Aung T., Medeiros F.A. (2014). The Pathophysiology and Treatment of Glaucoma: A Review. JAMA.

[46] Biousse V., Newman N. (2014). Retinal and Optic Nerve Ischemia. CONTIN. Lifelong Learn. Neurol..

[47] Karti O., Saatci I., Saatci A.O. (2025). Vascular supply of the eye: Clinical anatomy. Med. Hypothesis Discov. Innov. Ophthalmol..

[48] Strickland R.G., Garner M.A., Gross A.K., Girkin C.A. (2022). Remodeling of the Lamina Cribrosa: Mechanisms and Potential Therapeutic Approaches for Glaucoma. Int. J. Mol. Sci..

[49] Downs J.C., Girkin C.A. (2017). Lamina Cribrosa in Glaucoma. Curr. Opin. Ophthalmol..

[50] Albon J., Purslow P.P., Karwatowski W.S.S., Easty D.L. (2000). Age related compliance of the lamina cribrosa in human eyes. Br. J. Ophthalmol..

[51] Zhavoronkov A., Kanherkar R.R., Izumchenko E., Teka M., Cantor C., Manaye K., Sidransky D., West M.D., Makarev E., Csoka A.B. (2016). Pro-fibrotic pathway activation in trabecular meshwork and lamina cribrosa is the main driving force of glaucoma. Cell Cycle.

[52] Wang B., Lucy K.A., Schuman J.S., Sigal I.A., Bilonick R.A., Lu C., Liu J., Grulkowski I., Nadler Z., Ishikawa H. (2018). Tortuous Pore Path Through the Glaucomatous Lamina Cribrosa. Sci. Rep..

[53] Girkin C.A., Walker E., Gardiner S.K., Hallaj S., Murillo K., Jiravarnsirikul A., Weinreb R.N., Liebmann J.M., Zangwill L.M., Fazio M.A. (2026). Glaucomatous Remodeling of the Lamina Cribrosa: Association with Visual Field Progression. Investig. Ophthalmol. Vis. Sci..

[54] Chuangsuwanich T., Nongpiur M.E., Braeu F.A., Prasad S.C., Tun T.A., Thiéry A., Perera S., Ho C.L., Buist M., Barbastathis G. (2026). AI to Identify Strain-Sensitive Regions of the Optic Nerve Head Linked to Functional Loss in Glaucoma. Investig. Ophthalmol. Vis. Sci..

[55] Fraser C.L. (2014). Obstructive sleep apnea and optic neuropathy: Is there a link?. Curr. Neurol. Neurosci. Rep..

[56] Kaelin W.G., Ratcliffe P.J. (2008). Oxygen Sensing by Metazoans: The Central Role of the HIF Hydroxylase Pathway. Mol. Cell.

[57] Feng X., Zhang W., Wan T., Jiao K., Zhang L., Li C., Xiao L. (2025). Astrocytes from lamina cribrosa are involved in the autoregulatory function of optic nerve head vessels in vitro. Front. Mol. Biosci..

[58] Chidlow G., Wood J.P.M., Casson R.J. (2017). Investigations into Hypoxia and Oxidative Stress at the Optic Nerve Head in a Rat Model of Glaucoma. Front. Neurosci..

[59] Jassim A.H., Nsiah N.Y., Inman D.M. (2022). Ocular Hypertension Results in Hypoxia within Glia and Neurons Throughout the Visual Projection. Antioxidants.

[60] Jassim A.H., Fan Y., Pappenhagen N., Nsiah N.Y., Inman D.M. (2021). Oxidative Stress and Hypoxia Modify Mitochondrial Homeostasis During Glaucoma. Antioxid. Redox Signal..

[61] Tezel G., Wax M.B. (2004). Hypoxia-inducible factor 1alpha in the glaucomatous retina and optic nerve head. Arch. Ophthalmol..

[62] Reszeć J., Zalewska R., Bernaczyk P., Chyczewski L. (2012). HIF-1 expression in retinal ganglion cells and optic nerve axons in glaucoma. Folia Histochem. Cytobiol..

[63] Al Zoubi H., Riemer T., Simon R., Vilser W., Hasan S., Meller D., Augsten R., Hammer M. (2022). Optic disc blood perfusion and oxygenation in glaucoma. Graefe’s Arch. Clin. Exp. Ophthalmol..

[64] Eckert D.J., White D.P., Jordan A.S., Malhotra A., Wellman A. (2013). Defining phenotypic causes of obstructive sleep apnea. Identification of novel therapeutic targets. Am. J. Respir. Crit. Care Med..

[65] Peppard P.E., Young T., Palta M., Skatrud J. (2000). Prospective study of the association between sleep-disordered breathing and hypertension. N. Engl. J. Med..

[66] Logan A.G., Perlikowski S.M., Mente A., Tisler A., Tkacova R., Niroumand M., Leung R.S., Bradley T.D. (2001). High prevalence of unrecognized sleep apnoea in drug-resistant hypertension. J. Hypertens..

[67] Lv R., Liu X., Zhang Y., Dong N., Wang X., He Y., Yue H., Yin Q. (2023). Pathophysiological mechanisms and therapeutic approaches in obstructive sleep apnea syndrome. Signal Transduct. Target Ther..

[68] Won C.H.J., Qin L., Selim B., Yaggi H.K. (2018). Varying Hypopnea Definitions Affect Obstructive Sleep Apnea Severity Classification and Association with Cardiovascular Disease. J. Clin. Sleep Med..

[69] Hirotsu C., Haba-Rubio J., Andries D., Tobback N., Marques-Vidal P., Vollenweider P., Waeber G., Heinzer R. (2019). Effect of Three Hypopnea Scoring Criteria on OSA Prevalence and Associated Comorbidities in the General Population. J. Clin. Sleep Med..

[70] Lavie L. (2015). Oxidative stress in obstructive sleep apnea and intermittent hypoxia—Revisited—The bad ugly and good: Implications to the heart and brain. Sleep Med. Rev..

[71] Ryan S. (2018). Mechanisms of cardiovascular disease in obstructive sleep apnoea. J. Thorac. Dis..

[72] Garvey J.F., Taylor C.T., McNicholas W.T. (2009). Cardiovascular disease in obstructive sleep apnoea syndrome: The role of intermittent hypoxia and inflammation. Eur. Respir. J..

[73] Lurie A. (2011). Endothelial dysfunction in adults with obstructive sleep apnea. Adv. Cardiol..

[74] Kario K., Hettrick D.A., Prejbisz A., Januszewicz A. (2021). Obstructive Sleep Apnea-Induced Neurogenic Nocturnal Hypertension: A Potential Role of Renal Denervation?. Hypertension.

[75] Alomri R.M., Kennedy G.A., Wali S.O., Alhejaili F., Robinson S.R. (2021). Association between nocturnal activity of the sympathetic nervous system and cognitive dysfunction in obstructive sleep apnoea. Sci. Rep..

[76] Ohga E., Nagase T., Tomita T., Teramoto S., Matsuse T., Katayama H., Ouchi Y. (1999). Increased levels of circulating ICAM-1, VCAM-1, and L-selectin in obstructive sleep apnea syndrome. J. Appl. Physiol..

[77] Galassi F., Giambene B., Varriale R. (2011). Systemic vascular dysregulation and retrobulbar hemodynamics in normal-tension glaucoma. Investig. Ophthalmol. Vis. Sci..

[78] Sforza E., Roche F. (2016). Chronic intermittent hypoxia and obstructive sleep apnea: An experimental and clinical approach. Hypoxia.

[79] Carlos K., do Prado G.F., Tengan C.H. (2025). The Role of Mitochondria in Obstructive Sleep Apnea: Implications for the Upper Airway Muscles. Int. J. Mol. Sci..

[80] Yang Q., Wang Y., Feng J., Cao J., Chen B. (2013). Intermittent hypoxia from obstructive sleep apnea may cause neuronal impairment and dysfunction in central nervous system: The potential roles played by microglia. Neuropsychiatr. Dis. Treat..

[81] Bendel R.E., Kaplan J., Heckman M., Fredrickson P.A., Lin S.C. (2008). Prevalence of glaucoma in patients with obstructive sleep apnoea—A cross-sectional case-series. Eye.

[82] Hashim S.P., Al Mansouri F.A., Farouk M., Al Hashemi A.A., Singh R. (2014). Prevalence of glaucoma in patients with moderate to severe obstructive sleep apnea: Ocular morbidity and outcomes in a 3 year follow-up study. Eye.

[83] Bagabas N., Ghazali W., Mukhtar M., AlQassas I., Merdad R., Maniyar A., Almarzouki N., Afreen H., Badeeb O., Wali S. (2019). Prevalence of Glaucoma in Patients with Obstructive Sleep Apnea. J. Epidemiol. Glob. Health.

[84] Lin P.W., Friedman M., Lin H.C., Chang H.W., Wilson M., Lin M.C. (2011). Normal tension glaucoma in patients with obstructive sleep apnea/hypopnea syndrome. J. Glaucoma.

[85] Yu B.E., Cheung R., Hutnik C., Malvankar-Mehta M.S. (2021). Prevalence of Obstructive Sleep Apnea in Glaucoma Patients: A Systematic Review and Meta-Analysis. J. Curr. Glaucoma Pract..

[86] Bilgin G. (2014). Normal-tension glaucoma and obstructive sleep apnea syndrome: A prospective study. BMC Ophthalmol..

[87] Cheong A.J.Y., Wang S.K.X., Woon C.Y., Yap K.H., Ng K.J.Y., Xu F.W.X., Alkan U., Ng A.C.W., See A., Loh S.R.H. (2023). Obstructive sleep apnoea and glaucoma: A systematic review and meta-analysis. Eye.

[88] Garcıa-Sanchez A., Villalaın I., Asencio M., Garcıa J., Garcıa-Rio F. (2022). Sleep apnea and eye diseases: Evidence of association and potential pathogenic mechanisms. J. Clin. Sleep Med..

[89] Shi Y., Liu P., Guan J., Lu Y., Su K. (2015). Association between Glaucoma and Obstructive Sleep Apnea Syndrome: A Meta-Analysis and Systematic Review. PLoS ONE.

[90] Liu S., Lin Y., Liu X. (2016). Meta-Analysis of Association of Obstructive Sleep Apnea with Glaucoma. J. Glaucoma.

[91] Huon L.K., Liu S.Y.C., Camacho M., Guilleminault C. (2016). The association between ophthalmologic diseases and obstructive sleep apnea: A systematic review and meta-analysis. Sleep Breath..

[92] Zoh Y., Yun J.M. (2025). Association between obstructive sleep apnea and glaucoma. Korean J. Fam. Med..

[93] Tolosa-Tort C., Poza-Martin E., Garcia-Feijoo J., Mendez-Hernandez C. (2024). Study of the impact of the vascular systemic risk factors on peripapillary vascular density by optical coherence tomography angiography. Graefe’s Arch. Clin. Exp. Ophthalmol..

[94] Bussan K.A., Stuard W.L., Mussi N., Lee W., Whitson J.T., Issioui Y., Rowe A.A., Wert K.J., Robertson D.M. (2022). Differential effects of obstructive sleep apnea on the corneal subbasal nerve plexus and retinal nerve fiber layer. PLoS ONE.

[95] Zengin M.O., Tuncer I., Karahan E. (2014). Retinal nerve fiber layer thickness changes in obstructive sleep apnea syndrome: One year follow-up results. Int. J. Ophthalmol..

[96] Sun C.L., Zhou L.X., Dang Y., Huo Y.P., Shi L., Chang Y.J. (2016). Decreased retinal nerve fiber layer thickness in patients with obstructive sleep apnea syndrome: A meta-analysis. Medicine.

[97] Ferrandez B., Ferreras A., Calvo P., Abadia B., Marin J.M., Pajarin A.B. (2016). Assessment of the retinal nerve fiber layer in individuals with obstructive sleep apnea. BMC Ophthalmol..

[98] Wang J.S., Xie H.T., Jia Y., Zhang M.C. (2016). Retinal nerve fiber layer thickness changes in obstructive sleep apnea syndrome: A systematic review and Meta-analysis. Int. J. Ophthalmol..

[99] Shiba T., Takahashi M., Sato Y., Onoda Y., Hori Y., Sugiyama T., Bujo H., Maeno T. (2014). Relationship between severity of obstructive sleep apnea syndrome and retinal nerve fiber layer thickness. Am. J. Ophthalmol..

[100] Cai X., Wu X., Chen H., Lu X., Zhang C., Liu Y., Zhang X., Long D. (2025). Assessment of Retinal Perfusion and Vascular Density in Obstructive Sleep Apnea Syndrome with Swept-Source Optical Coherence Tomography Angiography. J. Sleep Res..

[101] Zhao X.J., Yang C.C., Zhang J.C., Zheng H., Liu P.P., Li Q. (2016). Obstructive Sleep Apnea and Retinal Nerve Fiber Layer Thickness: A Meta-analysis. J. Glaucoma.

[102] Wang W., He M., Huang W. (2017). Changes of Retinal Nerve Fiber Layer Thickness in Obstructive Sleep Apnea Syndrome: A Systematic Review and Meta-Analysis. Curr. Eye Res..

[103] Kargi S.H., Altin R., Koksal M., Kart L., Cinar F., Ugurbas S.H., Ayoglu F. (2005). Retinal nerve fibre layer measurements are reduced in patients with obstructive sleep apnoea syndrome. Eye.

[104] Teberik K., Eski M.T., Balbay E.G., Kaya M. (2018). Evaluation of Intraocular Pressure, Corneal Thickness, and Retinal Nerve Fiber Layer Thickness in Patients with Obstructive Sleep Apnea Syndrome. Pak. J. Med. Sci..

[105] Fan Y.Y., Su W.W., Liu C.H., Chen H.S.L., Wu S.C., Chang S.H.L., Chen K.J., Wu W.C., Chen N.H., Li H.Y. (2019). Correlation between structural progression in glaucoma and obstructive sleep apnea. Eye.

[106] Devi T.S., Agrawal A., Gupta N., Gupta R., Samanta R., Nishant P. (2023). Retinal Nerve Fiber Layer Thickness in Patients with Obstructive Sleep Apnea. J. Glaucoma.

[107] Ngoo Q.Z., Nazihatul Fikriah A., Baharudin A., Wan Hazabbah W.H. (2021). Evaluation of Retinal Nerve Fiber Layer Thickness and Optic Nerve Head Parameters in Obstructive Sleep Apnoea Patients. Korean J. Ophthalmol..

[108] Xin C., Zhang W., Wang L., Yang D., Wang J. (2015). Changes of visual field and optic nerve fiber layer in patients with OSAS. Sleep Breath..

[109] Chirapapaisan N., Likitgorn T., Pleumchitchom M., Sakiyalak D., Banhiran W., Saiman M., Chuenkongkaew W. (2016). Diurnal changes in retinal nerve fiber layer thickness with obstructive sleep apnea/hypopnea syndrome. Int. J. Ophthalmol..

[110] Ekinci M., Huseyinoglu N., Cagatay H.H., Ceylan E., Keles S., Gokce G. (2013). Is there a relationship between sleep apnea and central corneal thickness?. Curr. Eye Res..

[111] Moghimi S., Ahmadraji A., Sotoodeh H., Sadeghniat K., Maghsoudipour M., Fakhraie G., Latifi G., Nassiri N., Giaconi J.A. (2013). Retinal nerve fiber thickness is reduced in sleep apnea syndrome. Sleep Med..

[112] Çekiç B., Selçuk Ö.T., Toslak İ.E., Osma Ü., Eyigör H., Erol M.K. (2018). Does severe obstructive sleep apnea syndrome alter retrobulbar blood flow? A color Doppler ultrasound study. J. Med. Ultrason..

[113] Cristescu T.R., Mihălțan F.D. (2020). Ocular pathology associated with obstructive sleep apnea syndrome. Rom. J. Ophthalmol..

[114] Gutiérrez-Díaz E., Pérez-Rico C., De Atauri M.J., Mencía-Gutiérrez E., Blanco R. (2012). Evaluation of the visual function in obstructive sleep apnea syndrome patients and normal-tension glaucoma by means of the multifocal visual evoked potentials. Graefe’s Arch. Clin. Exp. Ophthalmol..

[115] Kadyan A., Asghar J., Dowson L., Sandramouli S. (2010). Ocular findings in sleep apnoea patients using continuous positive airway pressure. Eye.

[116] Karakucuk S., Goktas S., Aksu M., Erdogan N., Demirci S., Oner A., Arda H., Gumus K. (2008). Ocular blood flow in patients with obstructive sleep apnea syndrome (OSAS). Graefe’s Arch. Clin. Exp. Ophthalmol..

[117] Küçük B., Sırakaya E., Delibaş Ş. (2019). Posterior segment assessment in patients with obstructive sleep apnea syndrome. Sleep Breath..

[118] Lee S.S.Y., McArdle N., Sanfilippo P.G., Yazar S., Eastwood P.R., Hewitt A.W., Li Q., Mackey D.A. (2019). Associations Between Optic Disc Measures and Obstructive Sleep Apnea in Young Adults. Ophthalmology.

[119] Uslu H., Kanra A.Y., Sarac S. (2021). Structural assessment of the optic nerve in patients with obstructive sleep apnea syndrome: Case-control study. Eur. J. Ophthalmol..

[120] Asker S., Bulent Timucin O.B., Ursavas A., Emin Aslanci M.E., Baykara M., Erturk H., Asker M., Yilmaz S., Kaya D.T. (2013). Obstructive sleep apnea syndrome and blood flow to the eyes. East. J. Med..

[121] Carnero E., Bragard J., Urrestarazu E., Rivas E., Polo V., Larrosa J.M., Antón V., Peláez A., Moreno-Montañés J. (2020). Continuous intraocular pressure monitoring in patients with obstructive sleep apnea syndrome using a contact lens sensor. PLoS ONE.

[122] Chen R.I., Gedde S.J. (2023). Assessment of visual field progression in glaucoma. Curr. Opin. Ophthalmol..

[123] Lee G.A., Kong G.Y.X., Liu C.H. (2023). Visual fields in glaucoma: Where are we now?. Clin. Exp. Ophthalmol..

[124] Mills R.P. (1991). Statistical aids to visual field interpretation. J. Ocul. Pharmacol..

[125] Davanian A., Williamson L., Taylor C., Harrover A., Bollinger K., Chaudhary B., Taskar V., Lee T.J., Liu Y., Chen Q. (2022). Optical coherence tomography angiography and Humphrey visual field in patients with obstructive sleep apnea. J. Clin. Sleep Med..

[126] Salzgeber R., Iliev M.E., Mathis J. (2014). Do optic nerve head and visual field parameters in patients with obstructive sleep apnea syndrome differ from those in control individuals?. Klin. Monbl. Augenheilkd..

[127] Sergi M., Salerno D.E., Rizzi M., Blini M., Andreoli A., Messenio D., Pecis M., Bertoni G. (2007). Prevalence of normal tension glaucoma in obstructive sleep apnea syndrome patients. J. Glaucoma.

[128] Casas P., Ascaso F.J., Vicente E., Tejero-Garcés G., Adiego M.I., Cristóbal J.A. (2013). Retinal and optic nerve evaluation by optical coherence tomography in adults with obstructive sleep apnea-hypopnea syndrome (OSAHS). Graefe’s Arch. Clin. Exp. Ophthalmol..

[129] Singh M., Deokar K., Dutta S., Sinha B.P., Katoch C.D.S. (2025). Impact of positive airway pressure therapy on intraocular pressure in obstructive sleep apnea: A systematic review. Eur. J. Ophthalmol..

[130] Kongchan P., Banhiran W., Chirapapaisan N., Kasemsuk N. (2025). The effect of continuous positive airway pressure therapy on intraocular pressure in patients with OSA: A systematic review and meta-analysis. J. Clin. Sleep Med..

[131] Kiekens S., De Groot V., Coeckelbergh T., Tassignon M.J., Van De Heyning P., De Backer W., Verbraecken J. (2008). Continuous positive airway pressure therapy is associated with an increase in intraocular pressure in obstructive sleep apnea. Investig. Ophthalmol. Vis. Sci..

[132] Hirunpatravong P., Kasemsup T., Ayudhya W.N., Apiwattanasawee P. (2019). Long-term Effect of Continuous Positive Air Pressure Therapy on Intraocular Pressure in Patients with Primary Open-angle Glaucoma with Obstructive Sleep Apnea. J. Curr. Glaucoma Pract..

[133] Pépin J.L., Chiquet C., Tamisier R., Lévy P., Almanjoumi A., Romanet J.P. (2010). Frequent loss of nyctohemeral rhythm of intraocular pressure restored by nCPAP treatment in patients with severe apnea. Arch. Ophthalmol..

[134] Himori N., Ogawa H., Ichinose M., Nakazawa T. (2020). CPAP therapy reduces oxidative stress in patients with glaucoma and OSAS and improves the visual field. Graefe’s Arch. Clin. Exp. Ophthalmol..

[135] Chan J.H., Yeo B.S.Y., Lau W.K., Lau M.C., Koh J.H., Ng A.C.W., Goh L.C., Uataya M., Tin A., Toh S.T. (2026). Effect of Continuous Positive Airway Pressure on Intraocular Pressure in Patients with Obstructive Sleep Apnoea: A Systematic Review and Meta-Analysis. Clin. Exp. Ophthalmol..

